# Maternal sensitivity, infant limbic structure volume and functional connectivity: a preliminary study

**DOI:** 10.1038/tp.2015.133

**Published:** 2015-10-27

**Authors:** A Rifkin-Graboi, L Kong, L W Sim, S Sanmugam, B F P Broekman, H Chen, E Wong, K Kwek, S-M Saw, Y-S Chong, P D Gluckman, M V Fortier, D Pederson, M J Meaney, A Qiu

**Affiliations:** 1 Integrative Neuroscience Program, Singapore Institute for Clinical Sciences, Singapore, Singapore; 2 Department of Biomedical Engineering and Clinical Imaging Research Center, National University of Singapore, Singapore, Singapore; 3 Department of Psychological Medicine, Yong Loo Lin School of Medicine, National University of Singapore, National University Health System, Singapore, Singapore; 4 Department of Psychological Medicine, KK Women's and Children's Hospital, Duke-National University of Singapore, Singapore, Singapore; 5 Department of Maternal Fetal Medicine, KK Women's and Children's Hospital, Singapore, Singapore; 6 Department of Epidemiology, Saw Swee Hock School of Public Health, National University of Singapore, Singapore, Singapore; 7 Department of Obstetrics & Gynaecology, Yong Loo Lin School of Medicine, National University of Singapore, National University Health System, Singapore, Singapore; 8 Human Development, Singapore Institute for Clinical Sciences, Singapore, Singapore; 9 Liggins Institute, University of Auckland, Auckland, New Zealand; 10 Department of Diagnostic and Interventional Imaging, KK Women's and Children's Hospital, Singapore, Singapore; 11 Department of Psychology, University of Western Ontario, London, Ontario, Canada; 12 Department of Neurosciences, Ludmer Centre for Neuroinformatics and Mental Health, Douglas Mental Health University Institute, McGill University, Montreal, Quebec, Canada; 13 Sackler Program for Epigenetics and Psychobiology, Douglas Mental Health University Institute, McGill University, Montreal, Quebec, Canada

## Abstract

Mechanisms underlying the profound parental effects on cognitive, emotional and social development in humans remain poorly understood. Studies with nonhuman models suggest variations in parental care affect the limbic system, influential to learning, autobiography and emotional regulation. In some research, nonoptimal care relates to decreases in neurogenesis, although other work suggests early-postnatal social adversity accelerates the maturation of limbic structures associated with emotional learning. We explored whether maternal sensitivity predicts human limbic system development and functional connectivity patterns in a small sample of human infants. When infants were 6 months of age, 20 mother–infant dyads attended a laboratory-based observational session and the infants underwent neuroimaging at the same age. After considering age at imaging, household income and postnatal maternal anxiety, regression analyses demonstrated significant indirect associations between maternal sensitivity and bilateral hippocampal volume at six months, with the majority of associations between sensitivity and the amygdala demonstrating similar indirect, but not significant results. Moreover, functional analyses revealed direct associations between maternal sensitivity and connectivity between the hippocampus and areas important for emotional regulation and socio-emotional functioning. Sensitivity additionally predicted indirect associations between limbic structures and regions related to autobiographical memory. Our volumetric results are consistent with research indicating accelerated limbic development in response to early social adversity, and in combination with our functional results, if replicated in a larger sample, may suggest that subtle, but important, variations in maternal care influence neuroanatomical trajectories important to future cognitive and emotional functioning.

## Introduction

The quality of parental care influences socio-emotional and cognitive development,^1, 2, 3, 4^ as well as mental health,^5^ although the degree to which normal variation in parental care influences neural development is less well understood. In contrast, there are well-documented effects of more extreme forms of childhood adversity, such as abuse and neglect, upon brain development and function.^6, 7^ In addition, the influence of variations in parental care that lie within the normal range upon neuronal development and behavior has been extensively studied in rodents,^8^ with similar neurodevelopmental findings in nonhuman primates.^9, 10, 11^ Nevertheless, studies of human child development do reveal the particular importance of variation in ‘maternal sensitivity' on developmental outcomes.^12, 13^ Maternal sensitivity refers to timely and accurate responsivity to situationally dependent infant signals, and is critical for the management of infant distress and the facilitation of exploration and autonomy.^14, 15^ Meta-analytic analyses confirm that maternal sensitivity predicts infant behavioral responses to potentially stressful situations,^16^ which are themselves used to qualitatively describe the mother–child attachment relationship^17^—a highly documented predictor of subsequent socio-emotional development and mental health.^18^ Likewise, sensitivity and/or secure mother–child attachment predict positive social relationships,^19, 20, 21^ enhanced cognitive abilities,^22, 23, 24, 25^ and inversely relate to internalizing difficulties^26^ including anxiety,^27^ and externalizing problems.^26^ Furthermore, enhancing sensitivity has a positive effect on child outcomes.^28^

Despite the compelling evidence for the importance of maternal sensitivity for child development, there are no magnetic resonance imaging (MRI) studies that directly examine effects on early-postnatal brain structure and function. Findings from three studies using the Parental Bonding Instrument^29^ to retrospectively assess the potential influence of variations in the quality of parental care suggest that poorer parental care during childhood and adolescence may directly and/or indirectly negatively impact hippocampal volume during adulthood and the elderly years.^30, 31, 32^ These findings are consistent with those from rodent studies, suggesting that normal variations in maternal care alter limbic system development and function.^33, 34^ However, the results of these human studies are compromised by both the use of self-reported retrospective assessment of parental care in adulthood and measures of neural structure in later life. Self-reported Parental Bonding Instrument scores may be biased, as the degree to which they correlate with likely experience assessed by an objective observer is affected by the degree to which participants idealize parents and dismiss the importance of attachment relationships.^35^ Moreover, retrospective accounts do not report specifically on the quality of mother–infant, as opposed to child, experience, nor on the timing of the parental influences. Furthermore, neuroanatomical assessment later in development may reflect potential confounds such as subsequent social and emotional experience.

Consistent with research examining childhood maltreatment,^36^ prospective studies with observer-rated variations in maternal care do suggest effects on amygdala and hippocampal volume in childhood, early adolescence and adulthood.^37, 38, 39^ That is, Moutsiana *et al.*^39^ have recently reported that secure infant–mother attachment relationships observed at 18 months predicted smaller amygdala volume in early adulthood, and Whittle *et al.*^40^ observed positive maternal behavior during early adolescence predicted subsequent attenuation in amygdala growth. With regard to the hippocampus, Luby *et al.*^38^ found maternal support during early childhood directly predictive of hippocampal volume among non-depressed school-age children, whereas Rao *et al.*^37^ found increased nurturance as assessed by the Home Observation for Measurement of the Environment (HOME) Inventory scale indirectly associated with hippocampal volume among young adolescents who had been prenatally exposed to cocaine. Unfortunately, it is difficult to determine why the direction of effects on the hippocampus differs as, in addition to differences in the participant pools, the studies also differed with regard to the specific forms of caregiving experience assessed, the timing of exposure and the timing of MRI assessment, with all of these factors likely to influence relations between experience and brain development.^36^

Moreover, the timing of MRI assessment is not just an important reflection of the duration of time, and presumably physiological change, since exposure. MRI provides a ‘snapshot' of the brain's structure at a specific stage of development, with recent work suggesting that human development may not be uniform, and is influenced by experience with adversity^41, 42, 43, 44^ and positive parenting.^40^ Studying the effects on limbic structures during infancy is important, as this period is characterized by rapid hippocampal and amygdala growth.^45^ For example, MRI studies indicate that the hippocampus rapidly changes within the first one^46^ to two years.^46, 47^ The hippocampus substantially increases in volume (that is, by 15–19%) from the first to second year,^46^ suggesting that alterations occurring within the first year may also impact the course of development for future growth.

Thus, although an effect of maternal sensitivity upon infant hippocampal and amygdala volume is expectable, the direction of the hypothesized association remains unclear. Past literature suggests that brain regions may be especially sensitive to insults during periods of rapid growth,^48^ and that adversity or stress hormone exposure may lead to decreased hippocampal volume,^36, 49^ with mixed findings concerning relations between adversity, glucocorticoids and amygdala volume;^50, 51, 52^ following this line of reasoning, increased sensitivity should directly relate to hippocampal volume, but may directly or indirectly relate to amygdala volume. In contrast, as both the hippocampus and amygdala show normative volumetric increases over early infancy,^45^ ideas concerning adversity and acceleration in development^42, 43, 44, 53, 54^ suggest that increased sensitivity should indirectly relate to both hippocampal and amygdala volumes.

To our knowledge, no studies have yet examined the potential effects of maternal behavior on hippocampal and amygdala structure over this early-postnatal period. In this report, we provide a novel determination of the association between variation in observed maternal sensitivity and infant hippocampal and amygdala volume assessed using structural MRI. Then, we explore the relation specifically between maternal sensitivity and, separately, hippocampal and amygdala functional connectivity assessed using resting-state functional MRI (fMRI) at 6 months of age. Resting-state fMRI enables a summary of complex patterns of brain functional organization, which can be examined in relation to maternal sensitivity in the level of hippocampal and amygdala functional networks.

## Materials and methods

### Participants

Participants were part of a larger prospective birth cohort study, Growing Up in Singapore Towards Healthy Outcomes (GUSTO).^55^ The GUSTO cohort consisted of pregnant Asian women attending the first trimester antenatal ultrasound scan clinic at the National University Hospital and KK Women's and Children's Hospital in Singapore. The parents were Singapore citizens or permanent residents of Chinese, Malay or Indian ethnic background. Birth outcome and pregnancy measures were obtained from hospital records. Socioeconomic status (household income) was extracted from survey questionnaires conducted as a part of a scheduled appointment during pregnancy. The GUSTO study was approved by the National Healthcare Group Domain Specific Review Board and the Sing Health Centralized Institutional Review Board, and all participating mothers provided informed consent.

Inclusion criteria for infants in the current research included an Apgar score of ⩾9, gestational age ⩾37 and <42 weeks, birth weight ⩾2500 and <4000 g, singleton birth and born to mothers with no pregnancy complications such as hypoglycemia, hypertension, preeclampsia, reported intrauterine growth retardation or gestational diabetes. In addition, included infants had usable data from: a 6-month structural and/or functional MRI session; a behavioral observation session occurring within ±2 weeks of their 6-month birthday; and a neonatal structural MRI scan. Four-hundred and thirty-four mother–infant dyads provided useable data from the 6-month behavioral assessment. One hundred and eighty-nine infants were recruited for the neuroimaging study shortly after birth. A subset of these infants was invited back for the 6-month imaging visit. Among those invited for the 6-month visit, 42 infants came back for the second MRI scan at 6 months of age, although at 6 months only 32 of these participants provided artifact-free structural MRI data, and only 24 of these participants provided artifact-free functional MRI data.

### Maternal sensitivity

A 15-min mother–child interaction was recorded as part of a 3-h laboratory visit when infants were 6 months of age (±2 weeks). The mother was asked to ‘interact or play' with her 6-month-old infant ‘as she normally would at home.' The room was equipped with a foldable chair, highchair and a mat, but no toys for the first 5 min. After 5 min, a standard set of attractive toys and books was brought into the room. Maternal sensitivity was assessed using the Revised Mini-A short form of the Maternal Behavioral Q-Sort-V (Mini-MBQS-V).^56, 57, 58^ The Mini-MBQS-V consists of 25 items, each representing different possible aspects of sensitive, and inversely, insensitive, maternal behavior during interaction with an infant. Coders sort the 25 items into piles of 5, ranging from 1 being ‘least like the mother' to 5 being ‘most like the mother.' Ratings are then correlated with that of a theoretically constructed prototypical sensitive mother to derive the global sensitivity score, ranging from −1 (very much unlike a prototypical sensitive mother) to 1 (very much similar to a prototypical sensitive mother). For example, if the mother's behavior is very similar to a ‘prototypically sensitive mother', coders might assign values of ‘5' to cards such as: ‘mother builds on the focus of the baby's attention' and ‘mother responds to the baby's distress and non-distress signals even when engaged in some other activity.' Likewise, when viewing a mother who is very similar to a prototypically sensitive mother, coders might assign values of ‘1' to cards describing insensitive behavior such as, ‘mother tends to tune out and not notice the infant's bids for attention' and ‘the content and pace of the interaction is set by the mother rather than the baby's response.' The two southeast Asian coders who scored the majority of the current study's cases were directly trained by the developers of the Mini-MBQS-V coding system (D Pederson and S Bento). Together the local coders were fluent in both English and the predominant mother-tongue languages of Singapore, with one coder fluent in both English and Tamil, whereas the other is fluent in English, Bahasa Melayu and Mandarin. Training included the scoring of western and Singaporean tapes. To ensure reliability within the current sample, coders double coded all recordings where mothers predominantly spoke in English (that is, 70% of the current sample) and achieved an intra-class correlation of *r*=0.937. In addition, at the time of writing, the two local coders had achieved an high interclass correlation (*r*=0.923) on roughly 15% of useable cases comprising these and other cases within the larger GUSTO sample (*n*=434).

### MRI acquisition

During the acquisition sessions, infants slept in a 1.5-Tesla GE scanner (GE Healthcare, Milwaukee, WI, USA) at the Department of Diagnostic and Interventional Imaging of the KK Hospital. No sedation was used, and precautions were taken to reduce exposure to MRI scanner noise. A neonatologist was present during each scan. A pulse oximeter was used to monitor heart rate and oxygen saturation through the entirety of the scans.

The imaging protocols were (i) fast spin-echo T2-weighted MRI (axial acquisition; TR=3500 ms; TE=110 ms; FOV=256 × 256 mm; matrix size=256 × 256; 50 axial slices with 2.0 mm thickness); (ii) fast spin-echo T2-weighted MRI (coronal acquisition; TR=3500 ms; TE=110 ms; FOV=256 × 256 mm; matrix size=256 × 256; 50 axial slices with 2.0 mm thickness); (iii) echo planar resting-state fMRI (axial acquisition; TR=2500 ms; TE=40 ms; FOV=192 × 192 mm; matrix size=64 × 64; 40 axial slices with 3.0 mm thickness, 120 volumes). The coronal T2-weighted MRI data were acquired parallel to the anterior–posterior axis of the hippocampus and only covered the temporal lobe. Each subject obtained two acquisitions of the axial T2-weighted MRI and one acquisition of the coronal T2-weighted MRI at baseline and follow-up. All brain scans were reviewed by a neuroradiologist (MVF). Images were analyzed blind to sensitivity ratings.

### Hippocampus and amygdala delineation

Within individual subjects two T2-weighted MRI acquisitions were first rigidly aligned and averaged to increase signal-to-noise ratio. In cases where only one scan was acquired, data from one scan were used in lieu of the average axial image. The skull of the averaged axial image was removed using the Brain Extraction Tool^59^ and used manual and automated segmentation of the hippocampus and amygdala. The delineation protocol for the neonatal and 6-month-old infant's brain is detailed elsewhere.^60^ Intra-class correlation coefficients for the manual segmentation were, respectively, 0.79 and 0.82 for the hippocampus of neonates and 6-month-old infants, and 0.77 and 0.80 for the amygdala of neonates and 6-month-old infants.

### Limbic system functional connectivity analysis

#### Preprocessing

The resting-state fMRI data were first processed with slice timing, motion correction, skull stripping, band-pass filtering (0.01–0.1 Hz) and grand mean scaling of the data. We only included subjects with framewise displacement (head motion characteristics, ranging from 0.05 to 0.47 in our sample) <0.5 as suggested by Power *et al.*^61^ However, three subjects showed sudden ‘jerk-like' head movements at the first three volumes. We manually removed these three volumes to create the new fMRI data sets for these three subjects that satisfied the head motion criteria. Within each subject, the six parameters of the head motion and cerebrospinal fluid and white matter signals were regressed out from the fMRI images. Finally, the fMRI images were aligned to the T2-weighted image.

#### Functional connectivity

To analyze the hippocampus and amygdala functional networks, we first computed the mean signals within the hippocampus/amgdala marks delineated from the T2-weighted image. We then calculated Pearson's correlation coefficients of the hippocampal/amygdala fMRI signal with the fMRI signals of the rest of the brain and converted them as *z*-score using Fisher's *z*-transformation. Finally, the *z*-score images were aligned to the 6-month-old infant atlas based on the nonlinear transformation obtained from large deformation diffeomorphic metric mapping.^62^ These images in the atlas space were used for statistical analysis.

### Statistical analysis

#### Sensitivity and limbic structure volumes

The relation between maternal sensitivity and 6-month right and left hippocampal/amygdala volumes were determined via separate regressions. In these regressions, postmenstrual age at the time of MRI was entered in the first block, whereas maternal sensitivity was entered in the second block. Six-month left/right hippocampal/amygdala volume served as the outcome.

In addition, to better determine the specificity of effects, we also examined relations between maternal sensitivity and potential confounders incuding: gestational age, birth weight, birth length, pre- and postnatal maternal state and trait anxiety, pre- and postnatal maternal depression, household income, maternal education, maternal age, ethnicity and infant gender. The examinations of sensitivity and potential confounds were conducted within the larger sample of participants who had similar inclusion/exclusion criteria. No significant associations were observed for the majority of potential confounders. However, household income significantly related to sensitivity (*r*=0.213, *P*=0.001, *n*=261) and we observed a similar marginal association between sensitivity and maternal education (*r*=0.115, *P*=0.058, *n*=271). Likewise, the relation between levels of maternal state anxiety when infants were 3 months of age and maternal sensitivity at 6 months approached marginal significance (*r*=−0.105, *P*=0.116, *n*=227). In addition, we examined the relation between maternal sensitivity and neonatal hippocampal/amygdala volume among cases with similar inclusion/exclusion criteria who attended the neonatal scan, as sensitivity is known to relate to mental states^16^ associated with adult physiology,^63^ which could influence fetal development.^64^ Using partial correlations that controlled for age at the neonatal scan, and examining 80 dyads, maternal sensitivity did not significantly relate to left (*r*=−0.173, *P*=0.128) nor right (*r*=−0.097, *P*=0.397) neonatal hippocampal volume, nor left (*r*=−0.006, *P*=0.956) nor right (*r*=0.071, *P*=0.536) neonatal amygdala volume.

On the basis of the results of our covariate analyses, we repeated our regression analyses as above, but (a) first entered both postmenstrual age at the time of MRI and household income, before entering maternal sensitivity and (b) in a subsample for whom postnatal state anxiety information was available, first entered both postmenstrual age at the time of MRI and postnatal anxiety, before entering maternal sensitivity. All volumetric tests report two-sided *P*-values.

#### Sensitivity and limbic system functional connectivity

Voxel-based analysis was examined to first (separately) determine the hippocampal/amygdala functional connectivity networks and then investigate the potential influence of maternal sensitivity on the hippocampal/amygdala functional connectivity using SPM8 (Wellcome Trust Center for Neuroimaging, University College London, UK). The fMRI *z*-score images were smoothed with a Gaussian kernel with full width at half maximum of 6 mm. Regression analysis was first applied to the smoothed *z*-score images to identify the hippocampus/amygdala functional networks, with age at the 6-month MRI session as a covariate. Regression analysis was then used to examine the associations between maternal sensitivity and hippocampus/amygdala functional networks, with age at the 6-month MRI session and family income as covariates. Given the small sample size and concerns regarding power, as well as the weaker correlation between postnatal anxiety and sensitivity, postnatal anxiety was not entered as a covariate in these exploratory functional analyses. Consistent with similar research,^65^ all statistical results at each voxel were thresholded at the level of significance (*P*<0.01), the size of each cluster was greater than 100 voxels, uncorrected for multiple comparisons.

## Results

Of the 42 infants who participated in the 6-month MRI session, 32 participants had acceptable 6-month structural MRI data. Of these, 8 (25%) were excluded due to perinatal or pregnancy risks (for example, hypertensive pregnancy, prematurity, gestational diabetes and so on). Of the remaining 24 infants with usable 6-month volumetric data, in two cases the behavioral recording was corrupted—one was sick on the day of the behavioral assessment and one took multiple breaks during the testing procedure. Thus analyses examining the relation between maternal sensitivity and limbic structure volume included data from 20 infants who had income data, both neonatal and 6-month structural MRI data, sensitivity data and satisfied the above subject selection criteria.

Twenty-four infants had usable 6-month functional MRI data. Of these, four (17%) were excluded due to perinatal or pregnancy risks. Of the remaining 20 infants with usable 6-month functional data, two had corrupted behavioral data recordings, and one took multiple breaks during the testing procedure. Therefore, the analyses regarding the relation between maternal sensitivity and limbic structure functional connectivity included 17 subjects who had income data, 6-month sensitivity and resting-state MRI data, and satisfied the above subject selection criteria. Thus, these participants represent a smaller subsample than those included in the above structural analyses, although there is considerable overlap between the structural and functional samples.

To ensure the representative nature of the sample, we compared the maternal sensitivity scores of the mothers of the 20 infants included in the structural MRI analyses (*M*=0.29, s.d.=0.45) with the 258 mothers who did not have infants taking part in MRI (*M*=0.20, s.d.=0.42), but who otherwise fulfilled the perinatal/birth outcome criteria used in this study. No significant differences in sensitivity scores were observed, *t*_(1,276)_=0.864, *P*=0.388.

### Limbic structure volumes

**Figure 1:**
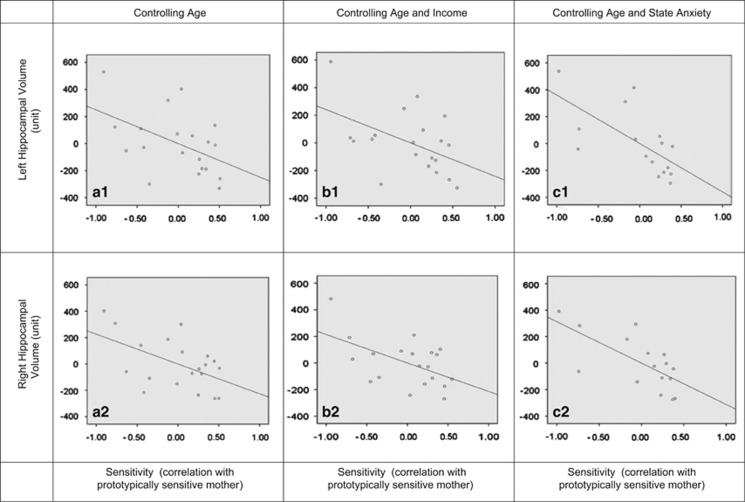
Partial regression plots of the association between maternal sensitivity and hippocampal volume. (**a**1) The association between maternal sensitivity and left hippocampal volume, controlling for age at MRI. (**a**2) The association between maternal sensitivity and right hippocampal volume, controlling for age at MRI. (**b**1) The association between maternal sensitivity and left hippocampal volume, controlling for age at MRI and household income. (**b**2) The association between maternal sensitivity and right hippocampal volume, controlling for age at MRI and household income. (**c**1) The association between maternal sensitivity and left hippocampal volume, controlling for age at MRI and maternal postnatal state anxiety at 3 months. (**c**2) The association between maternal sensitivity and right hippocampal volume, controlling for age at MRI and maternal postnatal state anxiety at 3 months. MRI, magnetic resonance imaging.

**Figure 2:**
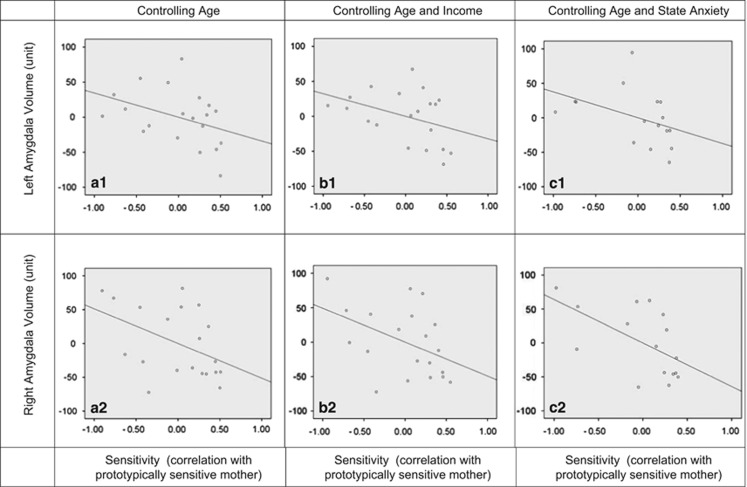
Partial regression plots of the association between maternal sensitivity and amygdala volume. (**a**1) The association between maternal sensitivity and left amygdala volume, controlling for age at MRI. (**a**2) The association between maternal sensitivity and right amygdala volume, controlling for age at MRI. (**b**1) The association between maternal sensitivity and left amygdala volume, controlling for age at MRI and household income. (**b**2) The association between maternal sensitivity and right amygdala volume, controlling for age at MRI and household income. (**c**1) The association between maternal sensitivity and left amygdala volume, controlling for age at MRI and maternal postnatal state anxiety at 3 months. (**c**2) The association between maternal sensitivity and right amygdala volume, controlling for age at MRI and maternal postnatal state anxiety at 3 months. MRI, magnetic resonance imaging.

The findings are summarized in Figures 1 and 2. After controlling for age, maternal sensitivity significantly predicted left (*B*=−0.490, *P*=0.037; **B**=−249.88, 95% confidence interval (CI)=−483.078 to −16.682) and right (*B*=−0.506, *P*=0.022; **B**=−226.045; 95% CI=−416.019 to −36.07) hippocampal volume at 6 months (see Figure 1). The associations were unlikely due to income as, after controlling for both age and family income, maternal sensitivity significantly predicted left (*B*=−0.474, *P*=0.045; **B**=−241.518, 95% CI=−477.348 to −5.688) and right (*B*=−0.479, *P*=0.023; **B**=−214.074, 95% CI=−394.702 to −33.446) hippocampal volume. Likewise, the associations were unlikely due to postnatal anxiety, as after controlling for age and maternal levels of state anxiety among the 16 cases for whom anxiety information was available, sensitivity significantly predicted left (*B*=−0.657, *P*=0.011; **B**=−360.011, 95% CI=−620.129 to −99.892) and right (*B*=−0.638, *P*=0.008; **B**=−312.220, 95% CI=−524.856 to −99.584) hippocampal volume. *Post hoc*-observed power analyses^66, 67^ indicated lower power for analyses involving the left hippocampus (that is, 0.51–0.71) than the right hippocampus (0.75–0.86).

The associations between sensitivity and hippocampal volume were unlikely due to outlying values, as in all cases, all data points fell within 3 s.d. of the regression slope, and all points fell within 3 s.d. from the mean for hippocampal volumes, sensitivity scores, age, income and postnatal anxiety. One case did have a Cook's distance exceeding the suggested 0.20 for this sample size both when income was not considered and when it was taken into account; when this case was removed from analyses, the results, although remaining consistent in their direction, no longer reached significance. Nevertheless, it is important to note that despite its Cook's distance, this case fell within 2 s.d. for the regression slope, which suggests that the large Cook's distance value may have been due to leverage, or the distance between this case and other cases in our small sample. Thus, given the case's biological feasibility and our small sample size, we believe it is best to retain this case in analyses. In addition, one (different) case had a Cook's distance exceeding the suggested 0.25 for the 16-dyad-smaller sample wherein postnatal anxiety was also considered; when this case was removed from analyses, the results controlling for age and anxiety and examining maternal sensitivity and hippocampal volume remained significant (left: *B*=−0.777, *P*=0.002; **B**=−475.927, 95% CI=−738.911 to −212.943; right: *B*=−0.780, *P*=0.001; **B**=−422.673, 95% CI=621.939 to −223.407).

The pattern of results was similar when we investigated the relation between maternal sensitivity and amygdala volume (see Figure 2), although these results generally failed to pass conventional significance levels. That is, maternal sensitivity marginally related to left (*B*=−0.389, *P*=0.102; **B**=−34.292, 95% CI=−76.104 to 7.52) and right (*B*=−0.451, *P*=0.057; **B**=−51.265, 95% CI=−104.288 to 1.759) amygdala volume. The results remained similar when controlling for income (left: *B*=−0.365, *P*=0.116; **B**=−32.166, 95% CI=−73.244 to 8.913; right: *B*=−0.433, *P*=0.068; **B**=−49.19, 95% CI=−102.526 to 4.147). Furthermore, when controlling for maternal postnatal state anxiety levels, the relation to the left amygdala remained nonsignificant (*B*=−0.386, *P*=0.144; **B**=−37.363, 95% CI=−89.409 to −14.684), whereas the relation to the right amygdala was significant (*B*=−0.552, *P*=0.034; **B**=−64.174, 95% CI=−122.572 to −5.777). Analyses were underpowered with *post hoc*-observed power analyses,^66, 67^ indicating power levels between 0.38–0.49 (left amygdala) and 0.44–0.57 (right amygdala). All values were within 3 s.d. of the regression slope, amygdala volumes, sensitivity, age, income and anxiety. For the most part, no points exceeded the suggested Cook's distance values; however, when anxiety was also considered one value exceeded the suggested Cook's distance for regressions involving the left amygdala, and two values exceeded the suggested value for regressions concerning the right amygdala. Nevertheless, when these values were respectively omitted from further analyses, results remained similar to the observed association between maternal sensitivity, controlling for age and postnatal anxiety on the left amygdala, failing to reach significance (*B*=−0.323, *P*=0.262; **B**=−27.256, 95% CI=−78.049 to −23.538), whereas the relation to the right amygdala remained significant (*B*=−0.692, *P*=0.005; **B**=−87.856, 95% CI=−142.348 to −33.364).

### Functional connectivity

**Figure 3:**
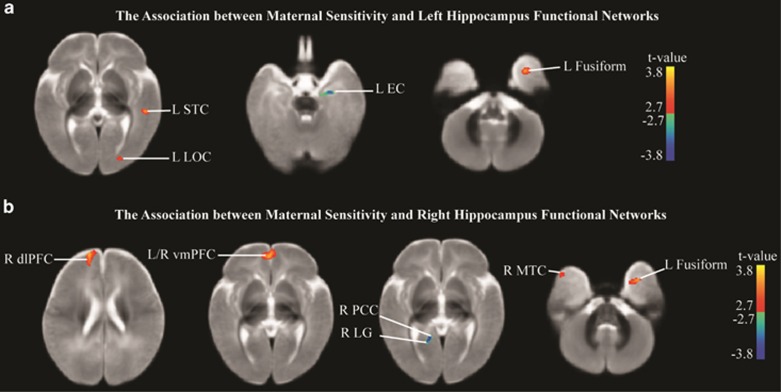
The association between maternal sensitivity and hippocampus functional connectivity. (**a**) The association between maternal sensitivity and left hippocampus functional connectivity. (**b**) The association between maternal sensitivity and right hippocampus functional connectivity. dlPFC, dorsolateral prefrontal cortex; EC, entorhinal cortex; L, left; LG, lingual gyrus; LOC, lateral occipital cortex; MTC, middle temporal cortex; PCC, posterior cingulate cortex; R, right; STC, superior temporal cortex; vmPFC, ventromedial prefrontal cortex.

**Figure 4:**
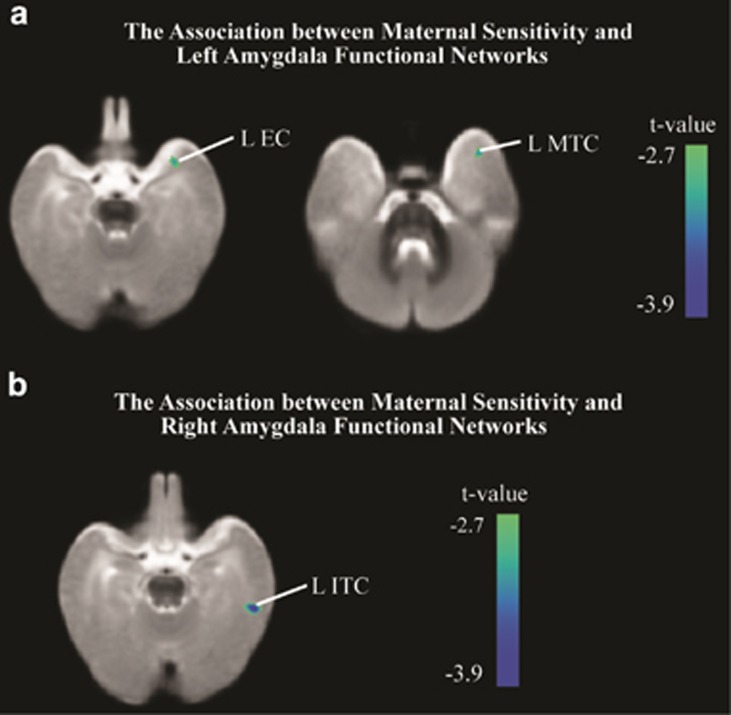
The association between maternal sensitivity and amygdala functional connectivity. (**a**) The association between maternal sensitivity and left amygdala functional connectivity. (**b**) The association between maternal sensitivity and right amygdala functional connectivity. EC, entorhinal cortex; ITC, inferior temporal cortex; L, left; MTC, middle temporal cortex.

After controlling for age and family income, maternal sensitivity significantly (that is, *P*<0.01, with clusters of over 100 voxels, uncorrected) positively predicted functional connectivity between the right hippocampus and regions associated with emotion regulation (that is, bilateral ventromedial prefrontal cortex (vmPFC)^68^), cognitive flexibility (that is, right dorsolateral prefrontal cortex (dlPFC)^69^) and social communication (that is, left fusiform^70^ and right middle temporal cortex^71^). Likewise, maternal sensitivity positively predicted left hippocampal connectivity to regions important to social communication (that is, the left fusiform^70^ and left superior temporal cortex^72^), as well as the left lateral occipital cortex. Maternal sensitivity negatively predicted connectivity with regions potentially important to autobiographical memory, such as the right lingual gyrus^73, 74^ and right posterior cingulate,^75^ as well as left hippocampal connectivity, with the left entorhinal cortex.^76^ Results are listed in Table 1 and displayed in Figures 3 and 4.

After controlling for age and family income, maternal sensitivity significantly (that is, *P*<0.01, with clusters of over 100 voxels, uncorrected) negatively predicted functional connectivity between the right amygdala and a region important to processing visual emotional stimuli (that is, the left inferior temporal cortex).^77^ In addition, sensitivity negatively predicted connectivity between the left amygdala and a region potentially important to autobiographical memory (that is, the left entorhinal cortex^76^), as well as the left middle temporal cortex).

## Discussion

Here, in our exploratory study, we focused on the impact of maternal care during infancy, a time when the hippocampus and amygdala undergo rapid development.^45, 46, 47^ Although our results cannot be considered representative of the larger population, and require replication in a larger sample where more rigorous statistical approaches may be applied, we nevertheless observed maternal sensitivity significantly (hippocampus) and marginally (amygdala) predictive of limbic structure volume in human infants. We also found preliminary evidence that maternal sensitivity was related to functional connectivity between the hippocampus and regions important to emotion regulation (that is, vmPFC^68^), cognitive flexibility (that is, dlPFC^69^), social communication (that is, fusiform,^70^ superior temporal cortex^72^ and middle temporal cortex^71^) and memory (that is, entorhinal cortex,^76^ lingual gyrus,^73, 74^ and posterior cingulate cortex^75^). Emotion regulation,^78, 79^ cognitive flexibility,^80, 81^ social behavior^19, 82^ and reported autobiographical memory^83, 84, 85, 86^ are all functions that vary with early mother–child relationships.

In contrast to findings concerning parenting and adolescent or adult hippocampal volume,^30, 32, 38^ our findings tentatively suggest that reduced maternal sensitivity associated with larger hippocampal volume during the infancy period. Although the direction of our volumetric finding may appear counterintuitive, this finding is consistent with the results of one study examining young adolescents.^37^ Moreover, although two meta-analyses of maltreatment-related post-traumatic stress disorder (PTSD) and pediatric hippocampal volume, as well as a recent meta-analysis examining maltreatment,^36^ suggest overall nil effects of childhood maltreatment on pediatric brain volumes, Tupler and De Bellis^87^ reported that among children with PTSD, childhood maltreatment was associated with a larger hippocampal volume, which in turn was associated with total level of risk for psychopathology on the Child Behavior Checklist. Likewise, Qiu *et al.*^60^ previously reported a positive association (***B***=0.992) specifically between right hippocampal growth within the first 6 months and postnatal maternal anxiety, which is linked to forms of care that may be nonoptimal for infant development.^88^ Indeed, these findings and those of the current study are consistent with an emerging view that social adversity in early life may increase the maturational rate of limbic structures, which mediate the activation of stress responses and emotional learning. Although mother–infant interactions in rodents, such as pup licking/grooming, dampen hypothalamic–pituitary–adrenal activity,^89^ stress accelerates the development of amygdala-dependent emotional learning in the rat, an effect that is mediated by stress-induced increases in glucocorticoids.^53, 54, 90^ Likewise, a remarkable translational study^44^ showed that early institutionalization and the associated absence of parental care was associated with accelerated maturation of fronto-amygdala connectivity. This effect was statistically mediated by cortisol levels, which were elevated in the previously institutionalized children (also see Gunnar *et al.*^91^), suggesting a parallel to the rodent models.

Thus, despite literature suggesting a negative influence of early-life stress on hippocampal volume,^92, 93^ the association between sensitive parenting and decreased limbic structure volume has precedence in both animal and human research. Indeed, there are many possible explanations for the discrepancies including the moderating influences of gender^30, 94^ and genotype,^95^ as well as the time course of limbic system development. The hippocampus develops rapidly in the first 2 years of life before its growth rate begins to plateau.^46, 47^ In later childhood to early adulthood (that is, 8–30 years), the hippocampus continues to grow,^96^ and its relation with age is influenced by pubertal status.^97^ Moreover, the relation with age is nonlinear, growing faster at earlier time points^96^ and following an inverted U-shaped curve, peaking at around 17 years of age.^98^ Thus, if stress exposure influences accelerated development, the timing of MRI acquisition may greatly influence whether relations between stress exposure and volume are direct, indirect or nil.

In addition to our structural findings, our results, although preliminary, also suggest environmental effects on functional connectivity apparent as early as 6 months of life. Although somewhat speculative, our small-sample findings suggest the possibility that increased maternal sensitivity associates with increased connectivity of pathways that dampen stress reactivity. The hippocampus is implicated in the regulation of stress responses,^99^ and both fMRI and positron emission tomography studies show that acute social stress leads to deactivation of the hippocampus and prefrontal regions.^100^ Furthermore, an fMRI experiment^100^ showed that the cortisol response to acute social stress was predicted by bilateral hippocampal deactivation. Although the hippocampus may inhibit cortisol responses to stress, other regions may be essential to assessing the nature and valence of the experience, to determine whether it is indeed stressful. The medial PFC is important in the interpretation of the personal relevance of concurrent and subsequent challenging situations. The left medial PFC exhibits enhanced fMRI activity when adults are asked to make self-referential judgments as well when these judgments are later remembered.^75^ fMRI studies demonstrate that the vmPFC, in conjunction with the hippocampus, is also important to context-based fear extinction.^68^ Milad *et al.*^68^ suggest that the vmPFC is important for associations concerning fear, but that the co-activation of the hippocampus is necessary to learn the conditions under which cues are no longer a valid signal of danger (that is, safety signals). Jin *et al.*^101^ found decreased positive connectivity between prefrontal and hippocampal regions among adults with PTSD, a disorder in which past trauma is undifferentiated from current context. In the current work, we found that maternal sensitivity was positively related to functional connectivity in infants between the hippocampus and bilateral vmPFC. Whether such variation in hippocampal–vmPFC connectivity mediates relations between maternal sensitivity and infants' perception of challenging situations and their accompanying stress responses^102, 103, 104^ may therefore be an interesting question for future research. Hippocampal deactivation and accompanying hypothalamic-pituitary-adrenal activity may influence additional prefrontal areas, including the dlPFC, important to executive functioning and attention.^69^ Here, our results suggest that maternal sensitivity was positively correlated with functional connectivity between the right hippocampus and right dlPFC. Behavioral and fMRI research in humans indicates that stress induction negatively impacts working memory, conceptualized via accuracy and reaction time during the 2-back (working memory) versus 0-back condition of an *n*-back test, and that stress induction decreases dlPFC activity during this test.^69^ Additional fMRI work with adults during an *n*-back test demonstrates the importance of dorsolateral–hippocampal coupling, such that decreased coupling relates to faster 2-back processing.^105^ In addition, Bernal-Casis *et al.*^106^ find consistent right dlPFC and left hippocampal decoupling during an *n*-back test across three study sites. Interestingly, recent findings demonstrating that sensitivity^80, 81^ and the closely related construct of secure mother–infant attachment^81^ predict enhanced childhood executive functioning, which requires flexibility in attention. Our sensitivity findings within this exploratory study, in conjunction with the aforementioned work concerning stress, and dlPFC–hippocampal connectivity, may begin to suggest a biological pathway through which early-life care affects later cognitive control. Variation in the flexibility of attention has additionally been considered important to the quality of attachment strategies,^107^ a developmental correlate of experience with sensitive care.^16^ Attachment strategies can be characterized as differential displays of attentional flexibility, with children previously experiencing sensitive care able to shift attentional demands based on environmental input, whereas those who have experienced less sensitive care may be constrained to following rigid strategies regardless of external experience.^107^

Beyond the associations between sensitivity and right hippocampal–prefrontal connectivity, we also observed preliminary evidence for positive relations between the hippocampus and regions less directly implicated in stress physiology and the management of emotion, but of potential importance to social behavior and communication. As noted, maternal sensitivity is a building block for mother–infant attachment relationships,^16^ which form a blueprint for social relationships throughout development.^83, 84, 85^ These blueprints have been repeatedly linked to thoughts and emotions important to social behavior, including friendship^108^ and intimacy.^109^ In adulthood, attachment representations, which are theoretically and empirically linked to early experiences with sensitive (versus insensitive) care,^83, 84, 85, 86^ correlate with electrophysiological correlates of face processing,^110^ and so may involve the superior temporal sulcus^111^ and/or the fusiform gyrus.^112^ Here, we observed sensitivity positively related to functional connectivity between the bilateral hippocampus and the left fusiform gyrus. Although limited work^113^ has examined the functional significance of such connectivity, a recent diffusion tensor imaging study revealed parallel bilateral pathways between the hippocampus and fusiform, with greater left laterality and potentially more myelination in human adults.^114^ Using functional connectivity methods, Miller and D'Espisito^113^ demonstrated right fusiform activity proceeding bilateral hippocampal activation during both encoding and retrieval phases of a facial memory task. These connectivity findings are consistent with accounts of hippocampal and fusiform co-activation during fMRI experiments examining encoding^115^ and response to novelty.^116^ Furthermore, in keeping with the notion that early experience affects adult social behavior, individual differences in emotionality,^117^ maltreatment^118, 119^ and trauma^120^ are all also predictive of hippocampal and fusiform co-activation during face-processing tasks. Likewise, in our exploratory study, maternal sensitivity predicted greater connectivity between the left hippocampus and the left middle temporal gyrus, a region also associated with experience with maltreatment,^121^ and, which, in coordination with the hippocampus, may support information processing relevant to social cognition.^122^ When viewing pictures of faces, both the left middle temporal gyrus and the left hippocampus show greater activation when biographical information is simultaneously retrieved than when it is not.^122^ In addition, both regions are also more responsive to ‘happy' as compared with neutral paired faces and voices.^123^ In addition, we also noted a positive association between maternal sensitivity and connectivity between the hippocampus and left superior temporal gyrus. Alterations in the activity levels of both the left superior temporal gyrus and hippocampus during a facial expression discrimination task have been observed in patients with Fragile X syndrome, which is partially characterized by social difficulties.^124^ Finally, we also observed a negative association between the right amygdala and the left inferior temporal cortex. Co-activation of the amygdala and inferior temporal cortex has been observed during the initial processing of emotional stimuli and is expected for environmentally salient visual stimuli, although inferior temporal activity continues even after the amygdala has habituated.^125^

In addition, we also observed associations between higher sensitivity and less connectivity between the limbic structures and structures involved in memory formation. Children who have experienced more sensitive maternal behaviors, and accordingly less insensitive behavior, in infancy are more likely than their counterparts to evidence rich autobiographical memories for childhood relationships in young adulthood. In specific, depending on the form of insensitive caregiving experienced, children and adults judged to have received high amounts of maternal insensitivity may be more likely to evidence little memory for childhood or excessive detail for early experiences.^83, 84, 85, 86, 126^ Here, in our exploratory study, we observed maternal sensitivity related to less connectivity between the hippocampus and regions important to memory. Namely, sensitivity negatively associated with functional connectivity between the left hippocampus and left entorhinal cortex, as well as the right hippocampus and the right lingual gyrus and right posterior cortex. Likewise, sensitivity also predicted less connectivity between the left amygdala and left entorhinal cortex. The entorhinal cortex may be important to autobiography, working as an interface between the hippocampus and frontal cortex to affect memory storage and retrieval.^76^ As one example, the entorhinal cortex co-activates with the hippocampus, during a delay period following the viewing of familiar faces that are subsequently accurately and confidently judged to have just been seen.^127^ The lingual gyrus shows enhanced activity during spatial^73^ and visual working memory,^74^ and altered resting-state lingual function differs in those likely to have experienced chronic perceived stress^128^ and may be associated with resilience to childhood maltreatment.^129^ The posterior cingulate cortex is also involved in memory, specifically showing increased activity in response to self-referential versus semantic judgments, with co-activation between the left hippocampus and posterior cingulate occurring during the encoding of referential material that is later remembered.^75^ Interestingly, Bluhm *et al.*^130^ find the functional connectivity between the right hippocampus and posterior cingulate is disrupted in women with PTSD, and suggest this alteration may explain related difficulties in distinguishing past trauma from the current environment. Moreover, Zhou *et al.*^131^ find that connectivity between the posterior cingulate cortex and right hippocampus/amygdala within days of trauma exposure predicts PTSD symptom severity. These results, in association with links between early attachment status and risk for dissociation in adolescence^132^ as well as adult disorganization in the face of loss or trauma,^84^ may imply that prior, early occurring environmental risk affects neuronal connectivity important to the integration of stressful or traumatic experience into adulthood. In sum, although speculative, the current results suggest that early experiences may shape connectivity patterns between neuroanatomical regions relevant for attachment and parenting-related memories later in life.

Understanding the influence of maternal sensitivity upon developmental trajectories and the influence of early variation in brain volume and function upon later memory formation, emotion and stress regulation will be an important avenue for future research using much larger samples. Of note, within the current analyses, one of the dyads, which, while still in the normal range, scored lowest on maternal sensitivity, may have had undue influence on the majority of hippocampal results, and in fact, relevant results did not remain significant when this case was removed. Thus, it is essential the current findings are replicated in larger-scale research, not only to ensure that they are not spurious, but also in order to better understand the nature of any association. That is, although the current small-sample research suggests a linear association, larger-scale research may be able to determine whether any effects of sensitivity on limbic development are more categorical in nature, with a greater influence being seen in cases at the lower end of the sensitivity spectrum. In low-risk samples, less than 40% of cases may be expected to exhibit a low degree of sensitive behavior.^133^ Thus, larger samples may be especially important to elucidate the potential influence of relatively extreme low sensitivity scores in a nonclinical group. Likewise, it will be essential to follow individuals over time to better understand whether the direction of the relation between caregiving adversity and hippocampal volume is dependent upon developmental stage, and whether this predicts subsequent risk. Considering both the rapid pace of hippocampal growth within the first 2 years of life,^46, 47^ and the results from this exploratory study suggesting that the infant hippocampus responds to relatively subtle forms of caregiving adversity with accelerated development, it is quite possible we would not have uncovered any relation between maternal behavior and hippocampal volume in older, low-risk children, although a lasting impact on functional connectivity may have still been detected. Indeed, in their recent work examining relations between infant attachment and adult brain volume, effects were observed on functional activity during an emotional task,^78^ and amygdala, but not, hippocampal volume.^39^ Despite this study's small sample size, a large effect on everyday variation in maternal care upon the 6-month-old hippocampii were identified, with similar, but, for the most part, only marginally significant effects on amygdala volume. Moreover, although our functional analyses with this sample did not pass multiple comparisons, here we found sensitivity related to the connectivity strength between the hippocampus and regions important to emotional and cognitive control, social functioning and memory. If replicated in a larger group, this work will suggest a clear need for widespread early parenting intervention programs. Although by definition maternal sensitive behavior can only be observed in the postnatal period, variation in sensitivity is known to associate with mental states^134^ that may impact hypothalamic-pituitary-adrenal activity^63^ and accordingly the uterine environment. However, in our analyses of potential confounding variables within the larger sample, maternal sensitivity did not significantly relate to infants' hippocampal volumes within the first 2 weeks of birth. Thus, a primary role of antenatal or solely genetic factors, which if fully explanatory, would likely have also affected the neonatal brain, is unlikely. Rather, our results tentatively suggest that the variation in species-relevant maternal care influences biological mechanisms to shape hippocampal development and functional connectivity. Given that roughly 30–50% of low-risk mothers are unlikely to consistently score high on measures of maternal sensitivity,^133, 135^ the current findings, if replicated in larger research, may help to explain a great deal of the variance in children's stress and emotional regulation within nonclinical groups. In sum, similar to the animal work, the current study suggests that species-specific subtle variation in parenting cues may impact the development of the infant brain in regions known to impact endocrine, cognitive and emotional functioning.

## Figures and Tables

**Table 1:** The association between maternal sensitivity and limbic structure functional connectivity

*Left hippocampus*		*Right hippocampus*	
*Positive associations*	*Negative associations*	*Positive associations*	*Negative associations*
L superior temporal cortex	L entorhinal cortex	R dorsolateral prefrontal cortex	R lingual gyrus
L fusiform		L/R ventromedial prefrontal cortex	R posterior cingulate cortex
L lateral occipital cortex		R middle temporal cortex	
		L fusiform	

*Left amygdala*		*Right amygdala*	
*Positive associations*	*Negative associations*	*Positive associations*	*Negative associations*
NA	L entorhinal cortex	NA	L inferior temporal cortex
	L middle temporal cortex		

Abbreviations: L, left; NA, not applicable; R, right.
